# Fungal Endophytes: Beyond Herbivore Management

**DOI:** 10.3389/fmicb.2018.00544

**Published:** 2018-03-23

**Authors:** Bamisope S. Bamisile, Chandra K. Dash, Komivi S. Akutse, Ravindran Keppanan, Liande Wang

**Affiliations:** ^1^State Key Laboratory of Ecological Pest Control for Fujian and Taiwan Crops, Fujian Agriculture and Forestry University, Fuzhou, China; ^2^College of Plant Protection, Fujian Agriculture and Forestry University, Fuzhou, China; ^3^Key Laboratory of Integrated Pest Management for Fujian and Taiwan Crops, Ministry of Agriculture, Fujian Agriculture and Forestry University, Fuzhou, China; ^4^Department of Entomology, Faculty of Agriculture, Sylhet Agricultural University, Sylhet, Bangladesh; ^5^International Centre of Insect Physiology and Ecology, Nairobi, Kenya

**Keywords:** fungal endophytes, biological control, entomopathogenic fungi, host plants protection, integrated pest management

## Abstract

The incorporation of entomopathogenic fungi as biocontrol agents into Integrated Pest Management (IPM) programs without doubt, has been highly effective. The ability of these fungal pathogens such as *Beauveria bassiana* and *Metarhizium anisopliae* to exist as endophytes in plants and protect their colonized host plants against the primary herbivore pests has widely been reported. Aside this sole role of pest management that has been traditionally ascribed to fungal endophytes, recent findings provided evidence of other possible functions as plant yield promoter, soil nutrient distributor, abiotic stress and drought tolerance enhancer in plants. However, reports on these additional important effects of fungal endophytes on the colonized plants remain scanty. In this review, we discussed the various beneficial effects of endophytic fungi on the host plants and their primary herbivore pests; as well as some negative effects that are relatively unknown. We also highlighted the prospects of our findings in further increasing the acceptance of fungal endophytes as an integral part of pest management programs for optimized crop production.

## Introduction

Endophytes are ubiquitous, forming associations with a diverse group of organisms throughout the plant kingdom and provide indirect defense for plants against herbivores (Hartley and Gange, 2009). Endophytes can exist in a host plant in the form of mutualistic root endophytes or plant-associated endophytes (Vega, 2008). They are plant-associated microorganisms that colonize and live part of their life cycle within a plant without causing harm or disease (such as lesions, retardation in growth, discoloration or chlorosis, etc.) to their host (Hardoim et al., 2015; Puri et al., 2016).

The tissues and organs of the host plants such as leaves, branches, stems, fruits, flowers, and roots are often colonized by fungal endophytes without showing visible symptoms (Saikkonen et al., 2006). Some fungal endophytes can also act as insect pathogenic agents by infecting lepidopterous larvae, aphids, thrips, and other cosmopolitan insects, which are of great concern in agriculture worldwide. They are known to infect specific hosts and pose little or no risk to non-target organisms or beneficial insects (Akutse et al., 2014).

There are various reports of possible artificial inoculation of plants with fungal entomopathogens to establish as plant endophytes artificially (Quesada-Moraga et al., 2009; Tefera and Vidal, 2009; Gurulingappa et al., 2010; Brownbridge et al., 2012; Parsa et al., 2013; Qayyum et al., 2015; Greenfield et al., 2016). Some previous reviews were focused on the role of endophytic fungi in plant herbivore management (Clay, 1989; Carroll, 1991, 1995; Breen, 1994; Saikkonen et al., 1998; Azevedo et al., 2000; Vega, 2008). Herein, we indicated that fungal endophytes such as *Beauveria bassiana* (Balsamo) Vuillemin (Ascomycota: Hypocreales) not only protect host plants from arthropod pests (Arnold and Lewis, 2005; Reddy et al., 2009; Akello and Sikora, 2012; Biswas et al., 2013), but also protect its hosts from diseases (Ownley et al., 2004, 2008b) and plant parasitic nematodes (Elmi et al., 2000; Sikora et al., 2007; Sikora et al., 2008), as well as enhance plant growth (Jaber and Enkerli, 2016; Jaber and Araj, 2017). Our review further summarized other effects of fungal endophytes on their host plants and primary pests. Furthermore, we highlighted the major sub-classes in which fungal endophytes can be classified based on criteria such as: their mode of reproduction, the source of nutrition, mode of transmission, symptoms expression in the hosts, the colonized plant parts, and their general morphology. We are of the opinion that the general knowledge of these findings would help to improve the application and overall adoption of fungal endophytes for pests and diseases management programs.

## Fungal Endophytes

Fungal endophytes have been reported as naturally occurring in several host plants (Saikkonen et al., 1998; Suryanarayanan, 2013). A single plant part (leaf, stem, or root) can contain different endophyte species (Cherry et al., 1999; Vega et al., 2008; Fürnkranz et al., 2012). Higher vascular plants have been found hosting endophytic fungi in a symbiotic plant-fungus interaction (Arnold and Lewis, 2005). The interaction is termed symbiotic, as endophytic fungi, in exchange for the nutrients derived from the host plants, provide benefits to their hosts. These fungal endophytes existing symbiotically within the colonized host plants are utilized as an indirect defense against herbivores (Kim et al., 2007, 2008; Powell et al., 2009; Quesada-Moraga et al., 2009).

Many fungi traditionally known as insect pathogens such as *Beauveria bassiana, Clonostachys rosea, Isaria farinosa*, and *Acremonium* sp. (now known as *Neotyphodium*) have been isolated as naturally occurring endophytes from asymptomatic plant tissues (Bills and Polishook, 1991; Cherry et al., 1999; Pimentel et al., 2006; Vega et al., 2008; Orole and Adejumo, 2009). In addition to the aforementioned, some many more endophytic entomopathogenic fungi were reported to have been re-isolated from colonized host plants after artificial inoculation. These include *Metarhizium anisopliae* (Fuller-Schaefer et al., 2005; Akello and Sikora, 2012; Greenfield et al., 2016); *B. bassiana* (Bing and Lewis, 1991, 1992; Wagner and Lewis, 2000; Parsa et al., 2013; Russo et al., 2015); *Fusarium oxysporum, Hypocrea lixii, Gibberella moniliformis*, and *Trichoderma asperellum* (Akello, 2012; Akello and Sikora, 2012; Akutse et al., 2013). According to the authors, artificially inoculated entomopathogenic fungi were successfully colonized at various degrees of endophytic colonization.

So far, using various artificial inoculation methods, successful colonization of several endophytic entomopathogenic fungi have been reported in wheat (*Triticum aestivum*) (Gurulingappa et al., 2010; Russo et al., 2015), common bean (*Phaseolus vulgaris*) (Akutse et al., 2013; Parsa et al., 2013, 2016), corn (*Zea mays*) (Bing and Lewis, 1991, 1993; Wagner and Lewis, 2000), tomato (*Lycopersicon esculentum*) (Ownley et al., 2008b; Qayyum et al., 2015), soybeans (*Glycine max*) (Russo et al., 2015), coffee (*Coffea* spp.) (Posada et al., 2007), opium poppy (*Papaver somniferum*) (Quesada-Moraga et al., 2006, 2009) cassava (*Manihot esculenta*) (Greenfield et al., 2016), sorghum (*Sorghum bicol*or) (Tefera and Vidal, 2009), cotton (*Gossypium hirsutum*) (Ownley et al., 2008b; Lopez et al., 2014; Lopez and Sword, 2015), and in some other economically important crops.

Generally, fungal endophytes are known to be beneficial to crop plants, while only few species can be pathogenic by causing diseases to the host after an incubation or latency period. Some species are neutral without offering benefit or posing harm to their hosts (Sikora et al., 2007, 2008). Petrini (1991) opined that some endophytic fungal species may exist as latent or inactive pathogens, but become active and reproduce under certain environmental conditions or when their host plants are stressed or grow old. This was supported by the findings of Alvarez-Loayza et al. (2011). Agrios (1988) on the other hand, described latent infections as the condition in which the host is infected without showing any symptoms. The pathogen is inactive or in latent state until symptoms are induced as a result of environmental changes, nutritional conditions of the host plant or the stage of maturity of the pathogen or the host.

There are speculations that, many thousands of endophytes useful to mankind are currently exiting but still unexplored due to limited research attention in this related field. However, with environmental contamination, deforestation, habitat fragmentation and biodiversity losses, many of these endophytes might be permanently lost before their value is explored (Kandalepas et al., 2015). There is, therefore, need to explore these biological agents reservoir not only for the management of arthropod pests and diseases, but also to elaborate on their diversity and other functions under different agro-ecological zones.

## Classification of Fungal Endophytes

Endophytes are diverse in taxonomy, but only few species have been isolated, identified and characterized to date (Hawksworth, 2001). Fungal endophytes can be classified broadly into ecological categories or otherwise, in terms of their diversity or functional roles. Based on these categories, they have been grouped into two major groups as clavicipitaceous and non-clavicipitaceous fungal endophytes. Clavicipitaceous fungal endophytes are mostly common in grasses, while the non-clavicipitaceous are predominant with vascular and non-vascular plant species (Rodriguez et al., 2009).

However, various authors have indicated the need to further classify these fungal endophytes to sub-classes based on different criteria such as: the host range, the mode of reproduction, the part of plant colonized, the mode of transmission, source of nutrition, and ability to express symptoms in the host plant (see Varma et al., 1999; Brem and Leuchtmann, 2001; Saikkonen et al., 2002; Rodriguez et al., 2009; Purahong and Hyde, 2011).

Fungal endophytes are classified **based on the mode of reproduction** as: sexual or asexual (Brem and Leuchtmann, 2001). For instance, the *Epichloë* endophytes have been divided into the genera *Epichloë* and *Neotyphodium* (formally *Acremonium*) that reproduces sexually and asexually respectively (Moon et al., 1999; Leuchtmann et al., 2000; Schardl and Craven, 2003).

They can as well be classified **based on the** **mode of transmission** in the host as: vertically–transmitted and horizontally–transmitted endophytes (Saikkonen et al., 2002). Vertically-transmitted endophytes are transferred directly from the host plants (parents) to their progenies (Saikkonen et al., 2002). True endophytes (such as the species belonging to the genus *Neotyphodium*) are mostly vertically transmitted through seeds from one plant to another (Hartley and Gange, 2009). When transmission is vertical through the host seeds, they are referred to as seed-transmitted endophytes (Dongyi and Kelemu, 2004; Bennett et al., 2008). Most *Epichloë* endophytes are seed-transmitted endophytes (Schardl et al., 2013). In addition, the paper of Quesada-Moraga et al. (2014) also reported vertical transmission of *B. bassiana* through seeds in opium poppy plants inoculated artificially via seed soaking.

On the other hand, horizontally-transmitted endophytes are transferred between different individuals in a given population. This mode of transmission is common with fungal endophytes that infect plants via airborne spores (Hartley and Gange, 2009). In this case, endophytes are usually multiplied via vegetative propagules, or transmission by spores in the case of spore-transmitted endophytes (Faeth and Fagan, 2002). Most woody and herbaceous plants harbor different species of unspecialized endophytic fungi. These fungal species which generally exhibit weak pathogenicity against insect herbivores are mostly transmitted horizontally (Higgins et al., 2007; Sieber, 2007).

Another classification is **based on the source of nutrition**, that is, whether nutrients are derived by the fungus from living or dead matter. Based on this, endophytes can be classified as necrotrophs, or as biotrophs. Biotrophic fungi are the types that develop and obtain nutrients within the tissue of a living host, while, necrotrophic fungi are the species that mortify the host cells in order to grow on the dead tissues (Kemen and Jones, 2012; Delaye et al., 2013). All endophytes are heterotrophs, unlike green plants that utilize CO_2_ directly for photosynthesis, they obtain carbon from the plants in the form of organic compounds (Pace, 1997). These fungi-plant interactions occur when endophytic fungi obtain their carbon supply from their hosts, and in exchange for the energy resources they derived from the host plants, provide benefits to the plant (Lekberg and Koide, 2005; Behie et al., 2012; Behie and Bidochka, 2014). However, as a result of periodic evolutionary and ecological changes, there is possibility of some fungal endophytes switching between the two lifestyles. That is, from biotrophic to necrotrophic lifestyle (Promputtha et al., 2007; Purahong and Hyde, 2011; Delaye et al., 2013). For instance, *Leptosphaeria maculans* occurring asymptomatic in healthy *Arabidopsis thaliana* plants became a necrotrophic pathogen when the plant was stressed (Junker et al., 2012).

**Based on the expression of infection**, endophytes are classified as symptomatic (expressing symptoms) and asymptomatic (symptomless) (Pinto et al., 2000). A good number of endophytic fungi infect above ground internal plant tissues without showing symptoms. Great attention is focused on these species of endophytes because they are ubiquitous and have vast diversity and many roles (Saikkonen et al., 2006; Arnold and Lutzoni, 2007). For instance, *Fusarium* spp. was identified as asymptomatic as it was confirmed to express no symptoms in cord roots of banana cultivar (*Pisang Awak* – *Musa* ABB) (Niere, 2001; Sikora et al., 2008). Although in some rare cases, symptomatic endophytes can be categorized as asymptomatic when the host plant is resistant to the fungi. However, as earlier stated, a change in environmental conditions could cause a sudden switch in the behavior of asymptomatic endophytic fungi. A clear example is the case of fungal species that were isolated as symptomless endophytes, yet, became pathogenic under changed environmental conditions (Delaye et al., 2013). To this end, we can reach a conclusion that, the age of the host plant harboring the fungus and the environmental conditions have a larger role to play in determining whether an endophytic fungus acts as a symptomless endophyte or otherwise as a symptom-producing plant pathogen (Saikkonen et al., 1998; Schulz and Boyle, 2005; Hyde and Soytong, 2008; Porras-Alfaro and Bayman, 2011).

**On the basis of the host plant part that is affected** by the fungal endophytes, they can be classified as root and foliar endophytes. The possibility of endophytic fungi to exhibit preferential tissue colonization within their colonized hosts has widely been reported. For instance, in a study conducted by Behie et al. (2015), *B. bassiana* and *Pochonia chlamydosporia* were reportedly localized within the stems and leaves, while *Metarhizium* spp. was mostly found within the plant roots. Several other previous findings have also reported the potential of endophytic fungi exhibiting localized endophytic colonization rather than colonizing the host plants systemically (Impullitti and Malvick, 2013; Yan et al., 2015). Thus, fungal endophytes that infect plant roots (as in the case of *Fusarium* spp., *Metarhizium* spp., *Piriformospora indica*, and *Glomus* spp.) are known as root endophytes (Varma et al., 1999; Wilberforce et al., 2003; Wyrebek et al., 2011). These groups of endophytes infect plant tissues from the rhizosphere (Skipp and Christensen, 1989). Other endophytes that invade stems and leaves of plants or species that are primarily localized to the foliar part of the plant are known as foliar endophytes (Meyling et al., 2011).

Fungal endophytes are generally identified based on their morphological features, they can be isolated from their host plant tissues and cultured in the most suitable growth media for morphological identification and classification (Clark et al., 1983). However, aside morphological characterization, there are suggestions that, isolation and molecular characterization of entomopathogenic endophytes are very imperative in order to expand the current data on entomopathogenic fungi (Lu et al., 2015). Molecular phylogenetic classification to identify endophytes can be carried out through amplifying and sequencing a small fragment of fungi DNA (Chen et al., 2015). This practice involves the analysis of nucleic acids and proteins to study the evolutionary relationships of fungal endophytes. A molecular phylogenetic relationship of *Epichloë typhina* with other clavicipitaceous endophytes also confirms the suggestion that fungal endophytes may coevolve with their host plants (Schardl et al., 1991).

## Host Plants Association With Fungal Endophytes Outcomes

Fungal endophytes have been confirmed to produce several beneficial effects to their host plants (Arnold et al., 2003; Rodriguez et al., 2009). The potential of entomopathogenic fungal endophytes exerting detrimental effects on the insect pests feeding on the plants colonized by these endophytic pathogens has been widely reported in several recent studies (Quesada-Moraga et al., 2009; Gurulingappa et al., 2011; Gange et al., 2012; Gathage et al., 2016; Resquín-Romero et al., 2016; Sánchez-Rodríguez et al., 2018). Most of these findings provided results that indicate that plants colonized by endophytes are protected from substantial damage, and plant pests feeding on such plants are less productive. The mechanism through which endophytic fungi reduce insect herbivore damage are numerous, some of the common measures include: reduction in the insect developmental rate (Akello and Sikora, 2012; Akutse et al., 2013), causing feeding deterrence (McGee, 2002; Vega, 2008), retardation of insect growth, reducing survival and oviposition (Lacey and Neven, 2006; Martinuz et al., 2012).

Reduction in plant damage caused by many insect pests has been reported in several crop plants following treatment with endophytic entomopathogenic fungi. For instance, reduction in poppy stem gall wasp (*Iraella luteipes*) (Hymenoptera: Cynipidae) damage in opium poppy treated with *B. bassiana* was found by Quesada-Moraga et al. (2009), reduced tunneling by lepidopteran larvae of European corn borer *Ostrinia nubilalis* Hübner (Lepidoptera: Pyralidae) and *Sesamia calamistis* Hampson (Lepidoptera; Noctuidae) was also reported in maize (Bing and Lewis, 1991; Lewis et al., 2001; Cherry et al., 2004). See also (Bing and Lewis, 1992; Wagner and Lewis, 2000).

Leckie (2002) reported a reduction in the damage caused by *Helicoverpa zea* (Lepidoptera; Noctuidae) in tomato following treatment with *B. bassiana* (see also Powell et al., 2009). *H. zea* was also reported to be controlled in cotton using *B. bassiana* and *Purpureocillium lilacinum* (Lopez and Sword, 2015). 50% mortality of all larval instars and reduced longevity of *Tuta absoluta* (Meyrick) (Lepidoptera: Gelechiidae) larvae fed with *B. bassiana* colonized tomato leaves was also recorded by Klieber and Reineke (2016). Qayyum et al. (2015) also found a reduction in *Helicoverpa armigera* damage of tomato plants. Posada et al. (2007) recorded a similar result with Coffee berry borer (*Hypothenemus hampei*) in coffee plant treated with *B. bassiana.* Akello et al. (2008a) found a similar reduction in larval survival and overall reduction in plant damage by banana weevil (*Cosmopolites sordidus*) (Coleoptera: Curculionidae). See also (Akello et al., 2008b).

Reduction in damage caused by the cotton aphid *Aphis gossypii* Glover (Hemiptera: Aphididae) and white jute stem weevil (*Apion corchori*) in cotton and white jute respectively was reported by Gurulingappa et al. (2010) and Biswas et al. (2013). In addition, in our previous study, we also found a similar reduction in damage caused by *Liriomyza huidobrensis* (Diptera: Agromyzidae) in *Vicia faba* and *P. vulgaris* (see Akutse et al., 2013). Some other reports on insect pests damage reduction following treatment of crops with entomopathogenic fungi are available (see Kim et al., 2008, 2010; Muvea et al., 2014; Resquín-Romero et al., 2016; Jaber and Araj, 2017; Rondot and Reineke, 2018; Sánchez-Rodríguez et al., 2018). Most of these studies have attributed the reduction in the damage by insect pests to the accumulation of mycotoxins in plant tissues (Gurulingappa et al., 2011). Clay and Schardl (2002) opined that the harmful effects of endophytic fungi on insect herbivores are due to the production of fungal metabolites.

Some previous studies have also indicated the possibility of using fungal endophytes and natural enemies such as parasitoids in combination for suppressing insect herbivore population and damage in plant. For instance, Jaber and Araj (2017) reported the possibility of using endophytic fungal entomopathogens, *B. bassiana* and *Metarhizium brunneum* in combination with the aphid endoparasitoid *Aphidius colemani* Viereck (Hymenoptera: Braconidae) for the management of the green peach aphid *Myzus persicae* Sulzer (Homoptera: Aphididae) in sweet pepper *Capsicum annum* L. (Solanaceae). Similarly, in one of our previous studies, we also reported the possibility of utilizing endophytic entomopathogenic fungi and either of the two leafminer parasitoids *Phaedrotoma scabriventris* (Hymenoptera: Braconidae) and *Diglyphus isaea* (Hymenoptera: Eulophidae) in combination for the management of pea leafminer *L. huidobrensis* in *V. faba* (Akutse et al., 2014) (see also Barker and Addison, 1996, 1997; Bultman et al., 2003; De Sassi et al., 2006).

However, some of the previous studies reported negative effect of fungal endophytes on the natural enemies parasitizing insects feeding on fungi-colonized plants (Bultman et al., 1997; Omacini et al., 2001; Faeth and Bultman, 2002; Kunkel and Grewal, 2003; Kunkel et al., 2004). The negative effects reported include reductions in growth, fecundity and adult survival of natural enemies (Omacini et al., 2001). The primary cause of these adverse effects is the transmission of mycotoxins across the food chain from the colonized plants through the insect pests to the parasitoids. A typical example is the case of *Neotyphodium coenophialum* which produces loline alkaloids that reduced the survival of the parasitoid *Euplectrus comstokii* introduced in tall fescue for the management of fall armyworm (*Spodoptera frugiperda*) (Bultman et al., 1997). Kunkel et al. (2004) also suggested that the negative effects of endophytic fungi on natural enemies may be due to the transfer of endophyte-produced toxins. There are also suggestions that the reduction in the size of insect herbivores feeding on endophyte-infected plants (Richmond et al., 2004) and possible reduction in nutritional value of the insects due to infection by endophytic entomopathogenic fungi may indirectly affect the natural enemies (Omacini et al., 2001).

Aside herbivore management, the potential of endophytic entomopathogenic fungi serving dual purpose biological control of both insects and plant pathogens has been reported (Ownley et al., 2004, 2008a; Griffin et al., 2006; Kim et al., 2007, 2010; Vega, 2008; Vega et al., 2009; Jaber and Salem, 2014; Jaber, 2015). Ownley et al. (2008b) reported that endophytic colonization of tomato and cotton seedlings through seed soaking in *B. bassiana* conidia protected the seedlings against plant pathogenic *Rhizoctonia solani* and *Pythium myriotylum.* In another study by Flori and Roberti (1993), basal rot of onion (a disease caused by *Fusarium oxysporum* f. sp. cepae) was significantly reduced following treatment of onion bulbs with *B. bassiana*. Similarly, disease incidence and severity of downy mildew – caused by *Plasmopara viticola* (Berk. and Curt.) Berl. and de Toni. was also significantly reduced in grapevine following colonization of leave tissues by *B. bassiana* (Jaber, 2015). The evidence of *B. bassiana* offering protection against plant viral pathogens is also available. Jaber and Salem (2014) found evidence of reduction in disease incidence and severity of *Zucchini yellow mosaic virus* (ZYMV) in *B. bassiana* inoculated squash plants.

In addition to pests and diseases management, fungi occurring in the host plants as endophytes provide other benefits to the colonized host. Established beneficial effects include: increasing plant growth (Lopez and Sword, 2015; Jaber and Enkerli, 2016), plant development and nutrients (nitrogen and phosphorus) uptake into plants (Behie et al., 2012; Behie and Bidochka, 2014) and improvement in overall plant hardiness (Khan et al., 2012), as well as, preventing colonization of the host by foreign parasitic organisms (Martinuz et al., 2012).

Endophytes colonize the host plant tissue hence creating a barrier that prevents foreign pathogenic organisms from colonizing the same host plant and consequently control phytopathogenic diseases (Moy et al., 2000). Endophytes are considered as primary sources of bioactive compounds, that not only serve as storehouse of unique bioactive secondary metabolites, such as alkaloids, saponins, tannins, phenolic acids, steroids, quinones and terpenoids, but also act as insect antagonist, antimicrobial, anticancer and many other important properties (Gouda et al., 2016). They can as well be referred to as biofertilizers because they serve as plant growth promoters that facilitate nutrient uptake not only through plant root system, but also through the transfer of the insect-derived nitrogen to plants. For example, *Metarhizium robertsii* infects and kills soil-born insects, produces fungal mycelia from the dead insects and thereafter, forms an endophytic association with the plant roots, hence enhancing nitrogen translocation (Behie et al., 2012). The findings from the study conducted by Behie and Bidochka (2014) indicated that each of the crop plants examined - haricot bean (*P. vulgaris*), wheat (*T. aestivum*), soybean (*G. max*), and switchgrass (*Panicum virgatum*), derived a substantial amount of nitrogen from the soil insects infected with entomopathogenic fungi. They opined that *M. robertsii* possibly supplied nitrogen to the crop plants in exchange for carbon. There is evidence that nitrogen uptake by plants in this plant-fungi-soil interaction may play a larger role in soil nitrogen cycling and insect pests’ infection.

Fungal endophytes also improve the colonized plant height, weight and other growth parameters are also influenced. Jaber and Enkerli (2017) reported an improvement in the height, fresh weight of shoots and roots of *V. faba* plants following artificial inoculation of *Beauveria brongniartii, B. bassiana* and *M. brunneum*. In another study, *B. bassiana* and *P. lilacinum* also increased the growth and dry biomass of colonized cotton plants (Lopez and Sword, 2015). Several other previous studies have also related improved plant growth with endophytic entomopathogenic fungi. See (Kabaluk and Ericsson, 2007; Elena et al., 2011; Sasan and Bidochka, 2012; Liao et al., 2014; Jaber and Enkerli, 2016; Jaber and Araj, 2017).

Endophytes also induce chemicals that impede the growth and development of other competitors, including pathogenic organisms (Clark et al., 1989), help plants not only to tolerate biotic stresses such as below-ground herbivory by nematodes and other root-feeding insects (Cosme et al., 2016), but also, abiotic stresses, including salt, drought or heat stresses (Khan et al., 2012). Endophytic fungi also indirectly enhance seed dispersal by ants. In a study by Knoch et al. (1993), seeds of Fescue (*Festuca arundinacea* L. Schreb) infected with *Acremonium coenophialum* (now known as *Neotyphodium coenophialum*) were protected against two primary seed harvesting ants – *Pogonomyrmex rugosus* and *Pogonomyrmex occidentalis*. The infected seeds were discarded after being collected by the ants and this periodic dispersal by the insects indirectly improves seed distribution.

However, not all endophytic fungi-plant associations protect plants from insect herbivores, only few species associations act as defense mutualisms. In some cases, the presence of fungal endophytes in a plant can result in higher rates of water loss in leaves and it has been recorded that some endomycorrhizae may increase pest damage by making their host plants more susceptible to the pest (Mueller et al., 2005).

The beneficial effects of fungal endophytes on the host plants and primary pests are numerous and not limited to those highlighted so far in this review. The suggestion that the subject cannot be discussed exhaustively cannot be far from the truth. Our opinion is, there might be other roles of fungal endophytes yet uncovered. However, it is of note that, few reports on certain harmful effects on the host plants are also available (see **Table 1** and **Supplementary Figure S1**). In the light of this, it is of note that, the relationship that exists between host plants and endophytes can be likened to a balanced antagonism, as the host derives both positive and negative effects depending on the environmental conditions (Saikkonen et al., 1998; Schulz et al., 1999). Hence, the environmental conditions could be said to distinguish a mutualistic endophyte from a pathogenic endophyte (Richardson, 2000; Schulz and Boyle, 2005).

**Table 1:** Effects of fungal endophytes on their colonized host plants and the primary pests.

S/N	Fungal endophyte effects	Reference
1	Fungal endophytes induce systemic resistance in the colonized plants. They could also be transferred vertically from parent plants to their offspring, hence providing same resistance for the next generation.	Saikkonen et al., 2002; Griffin et al., 2006; Ownley et al., 2008b
2	Fungal endophytes protect host plants against plant pathogens. This has been reported in *B. bassiana* and *Lecanicillium longisporum.*	Kim et al., 2007, 2008, 2010; Ownley et al., 2008b; Jaber and Salem, 2014; Jaber, 2015; Puri et al., 2016
3	Fungal endophytes have also been found to induce a reduction in insects feeding on endophytic colonized plants.	Knoch et al., 1993
4	Endophytic fungi alter the nutritional level of the colonized plant and utilize this in the production of secondary metabolites. Certain chemical defenses previously reported to have been mediated by the plant have recently been proven to be induced by endophytic fungi.	Rowan et al., 1986
5	Fungal endophytes improve tolerance of colonized plants to biotic stress such as root herbivory by plant parasitic nematodes.	Sikora et al., 2007, 2008; Cosme et al., 2016
6	Fungal endophytes assist the colonized host plants in providing protection against insect herbivores. They cause retardation of insect growth, developmental rate and adult survival rate.	Saikkonen et al., 2004; Arnold and Lewis, 2005; Lacey and Neven, 2006; Jallow et al., 2008; Kim et al., 2008, 2010; Muvea et al., 2014; Resquín-Romero et al., 2016; Jaber and Araj, 2017; Rondot and Reineke, 2018; Sánchez-Rodríguez et al., 2018
7	After successful colonization of the plant, certain fungal endophytes prevent colonization of the same plant by other foreign parasitic organisms. They produce chemicals that inhibit the growth of other pathogenic organisms and competitors.	Moy et al., 2000; Martinuz et al., 2012
8	Fungal endophytes cause deterrence, the potentials of repelling insects from feeding on colonized plants has been ascribed to fungal endophytes. The mechanism for deterrence has been linked to the changes in the chemical composition of the endophytically colonized plants.	Latch et al., 1985; Tanada and Kaya, 1993; Daisy et al., 2002; McGee, 2002
9	Fungi promote nutrients uptake in their colonized plants. The increase in phosphorus and nitrogen uptake has been reported	Behie et al., 2012; Behie and Bidochka, 2014
10	They improve the plant’s tolerance to abiotic stresses, such as salt, drought or heat stresses. Improvement in overall plant hardiness has also been reported as endophytic fungi effects.	Márquez et al., 2007; Hamilton and Bauerle, 2012; Junker et al., 2012; Khan et al., 2012
11	Fungal endophytes improve crop yield, plant growth, cell division and development. Improvement in crop yield and fresh weight of common bean and corn plants treated with *M. anisopliae*, *B. bassiana* and *H. lixii* has been reported. Also, *B. bassiana* and *M. brunneum* improved several growth parameters in sweet pepper (*C. annum*) and *V. faba.*	Kabaluk and Ericsson, 2007; Elena et al., 2011; Behie et al., 2012; Sasan and Bidochka, 2012; Behie and Bidochka, 2014; Liao et al., 2014; Lopez and Sword, 2015; Gathage et al., 2016; Jaber and Enkerli, 2016; Jaber and Araj, 2017; Jaber and Enkerli, 2017
12	Oviposition rate is reduced; fungal endophytes make plant herbivores sterile and less productive, through changes in the chemical composition or profiles of the host plants that deter oviposition of adult insects.	Schmidt and Osborn, 1993
13	Fungal endophytes also serve as reservoirs of novel bioactive compounds. They produce metabolites, antibiotics, bioactive volatile compounds (such as ammonia, lipids, alkyl pyrones, hydrogen cyanide, alcohols, ketones and esters).	Clark et al., 1989; Bills et al., 1992; Calhoun et al., 1992; White et al., 2003; Schardl et al., 2013
14	Endophytic fungi indirectly enhance seed dispersal by ants	Knoch et al., 1993
15	Aside from insects’ deterrence, there are also reports of fungal endophytes ability to deter vertebrate herbivores such as birds, rabbits and deer from feeding on fungal colonized plants.	Lekberg and Koide, 2005
16	Fungal endophytes (in the case of mycorrhizae) distribute nutrients within the surrounding plants and other mycorrhizae.	Franklin et al., 2014
**Some detrimental effects on host plants and natural enemies**		
17	Fungal endophytes build up the host defense system at the detriment of the reproductive potential. Certain fungal endophytes were found to render grasses partially sterile at the expense of their fungal reproductive structures.	Clay et al., 1989
18	In some cases, fungal endophytes when occurring in plant might increase transpiration rate in the leaves.	White et al., 1993
19	Some endo-mycorrhizae may increase herbivore damage in plants by making their host plants more susceptible to the insect pests.	Mueller et al., 2005
20	Fungal endophytes may indirectly protect insect pests against their natural enemies, by producing alkaloids such as ergovaline, loline, etc. that reduce the developmental rate and survival of natural enemies. Certain endophytic fungi-induced toxins mediate reduced susceptibility of insects to their natural enemies such as parasitoids and entomopathogenic nematodes.	Bultman et al., 1997; Omacini et al., 2001; Kunkel and Grewal, 2003; Kunkel et al., 2004


## Conclusion

Fungal endophytes provide protection for crop plants against insects attack. The ability to minimize attack from all kinds of insect pests; lepidopterous larvae, aphids and thrips, and other cosmopolitan insects has widely been reported. This has also been the primary aim of artificial inoculation of entomopathogenic fungi into economic crops to establish as endophytes.

Aside insect management, the potential of fungal endophytes in providing protection for plants against plant-parasitic nematodes and plant disease pathogens, enhancing host growth, promoting nutrient acquisition and improving tolerance to abiotic stresses, as well as enhancing resistance to mammalian herbivores have all been reported and clearly emphasized in this review.

Since fungal endophytes promote phosphorus, nitrogen and other essential nutrients uptake in the host plants, deep knowledge on this would assist organic and inorganic fertilizer users to ensure optimum usage. Moreover, the ability of fungal endophytes to improve the plant ability to tolerate heat stress, salt, drought and other abiotic stresses adds a new dimension to host plants–endophytes interactions, and could significantly be explored or used in agriculture not only to mitigate pests and diseases under climate change conditions, but also as an alternative approach to entomopathogenic fungi autodissemination in inundating application. That is, fungal endophytes could be a suitable replacement for entomopathogenic fungi which are normally applied as inundative sprays to offer short-term pest control. These entomopathogenic fungi when successfully established as endophytes in plants can offer long-term pests and diseases control.

The recent discoveries indicating that fungal endophytes provide other beneficial effects to their host plants aside mere protection against pests will go a long way in defining the huge importance of fungal endophytes in crop production for human sustainability. Also, we hope that this information would increase the effective use of fungal endophytes by exploring all their functional attributes as an integral part of the integrated pest management programs throughout the different agroecological zones worldwide.

## Author Contributions

LW designed the review outline. The manuscript was written by BB, KA, and CD. KA and RK reviewed the manuscript.

## Conflict of Interest Statement

The authors declare that the research was conducted in the absence of any commercial or financial relationships that could be construed as a potential conflict of interest.

## Supplementary Material

FIGURE S1 Fungi, host plants and primary pests interactions.
