# Supplementary figures and images for: Fungal Endophytes: Beyond Herbivore Management
Source: Front Microbiol. 2018 Mar 23;9:544. doi: 10.3389/fmicb.2018.00544 (PMC5876286; doi:10.3389/fmicb.2018.00544)

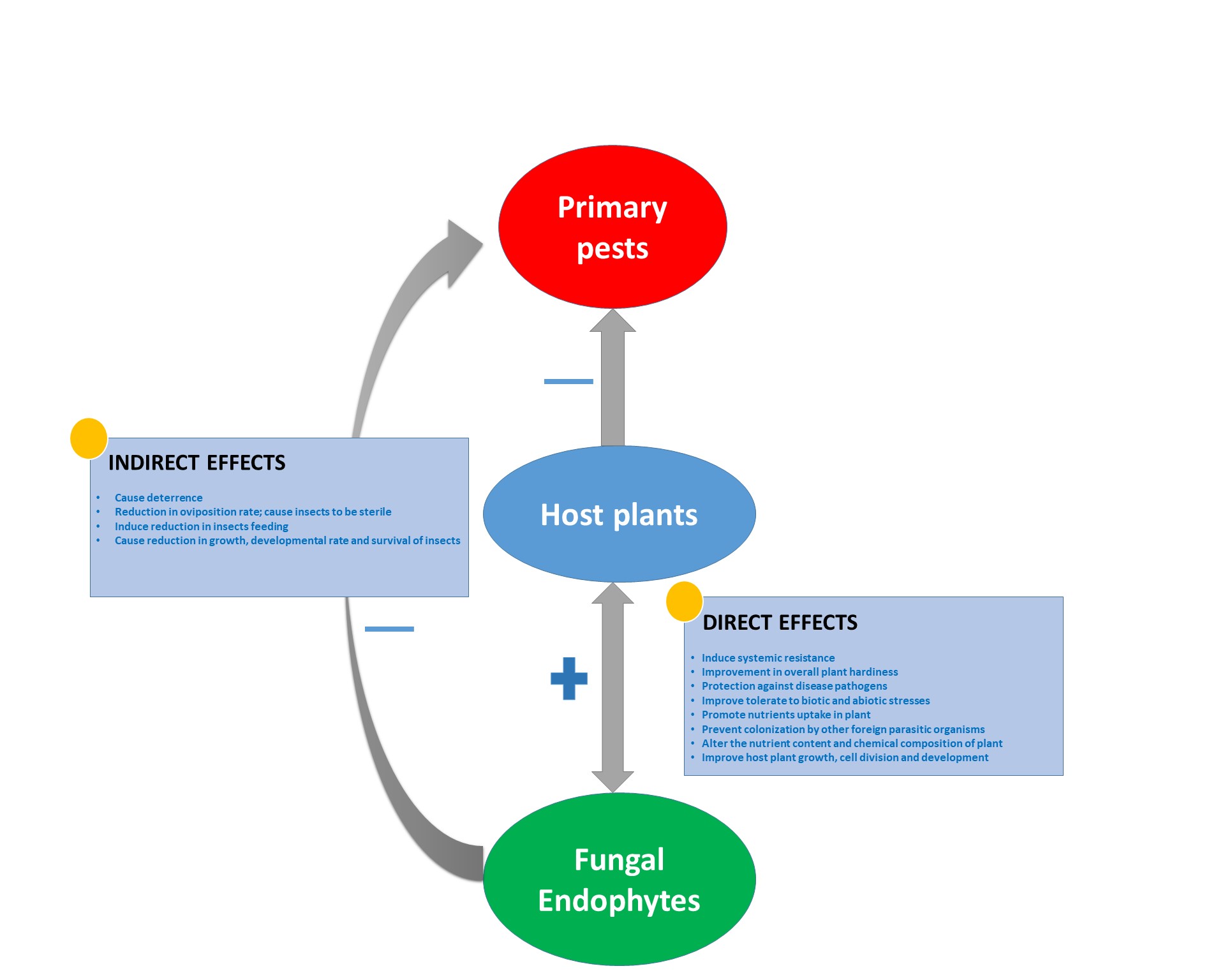

Supplement: FIGURE S1 — Fungi, host plants and primary pests interactions. [file Image_1.JPEG]
